# The Introduction of a Multidisciplinary Hip Fracture Pathway to Optimise Patient Care and Reduce Mortality: A Prospective Audit of 161 Patients

**DOI:** 10.2174/1874325001711010309

**Published:** 2017-04-20

**Authors:** Michael Shenouda, Zacharia Silk, Sarkhell Radha, Emer Bouanem, Warwick Radford

**Affiliations:** Department of Trauma and Orthopaedics, Chelsea & Westminster NHS Foundation Trust, London, UK

**Keywords:** Audit, Multidisciplinary Care, National Hip Fracture Database, Outcomes, Proximal femoral fracture

## Abstract

**Introduction:**

Hip fractures are a major cause of morbidity and mortality in the elderly. A new patient pathway was introduced in our institution to facilitate rapid preoperative assessment, acute physician involvement and early surgery for patients with hip fractures. We sought to assess its impact on patient care and outcomes.

**Materials and Methods:**

Prospective audit of 161 patients admitted with a proximal femoral fracture in the six months before (92 patients) and after (69 patients) implementation of the pathway. Data included: time to orthogeriatric assessment (TtG); time to surgery (TtS); length of hospital stay (LOS); return to original accommodation; inpatient mortality rate.

**Results:**

In the six months after introduction of the pathway, there was an increase in patients who received pre-operative medical assessment (85% after *vs.* 19% before, p=0.0001). Average TtG decreased (19 *vs.* 91 hours, p=0.0001), as did LOS (19.5 *vs.* 24.8 days, p=0.029) and mortality (4 *vs.* 14%, p=0.0336). There was an increase in patients returning to their original place of accommodation (80% *vs.* 57%, p=0.0069). There was a reduction in mean TtS (31 *vs.* 37 hours, p=0.0663), although this was not statistically significant.

**Discussion and Conclusions:**

Rapid medical optimisation and prompt surgery significantly improve outcomes in patients with hip fractures. By involving an acute medical team in patient care from the point of admission, we have significantly improved our inpatient mortality and increased the proportion of patients returning to their preoperative place of accommodation, thereby maintaining patient independence and reducing the financial and logistical burden on social care.

## INTRODUCTION

Hip fractures are already a major cause of morbidity and mortality in the elderly [1]. The current life-time risk of sustaining a hip fracture at 50 years of age is 17.5% amongst females and 6% amongst males [2]. Owing to an aging population, the annual incidence worldwide is expected to rise significantly [3], and is estimated to reach 6.26 million by 2050 [4]. At one year, mortality rates after hip fracture can be as high as 33% [5, 6], and is significantly associated with the number and severity of associated medical co-morbidities [7]. Furthermore, approximately 50% of patients will go on to lose their independence to varying degrees [8].

As a result, guidelines developed by the National Institute for Health and Clinical Excellence (NICE), British Orthopaedic Association (BOA) and British Geriatric Society (BGS) recommend early orthogeriatric assessment, prompt medical optimisation and surgery on the day of, or day after, admission [9]. On the back of Lord Darzi’s Next Stage Review, High Quality Care for All [10], these clinical standards were financially incentivised with the introduction of a Best Practice Tariff (BPT) in April 2010. To achieve the BPT, the collection and submission of data to the UK’s National Hip Fracture Database (NHFD) became mandatory, which has allowed it to effectively become one of the largest prospective clinical audits taking place in the United Kingdom.

In our institution, a novel multidisciplinary hip fracture pathway was developed to allow best practice to be applied, with the key aim being to ensure prompt pre-operative medical optimisation. The pathway was agreed by all involved parties, including emergency department staff, acute physicians, orthogeriatricians, anaesthetists, orthopaedic surgeons, and nursing staff on both the acute assessment unit (AAU) and orthopaedic wards. Implementation was led by our trauma lead nurse (EB). Amongst other key interventions, the primary admitting consultant was changed from an orthopaedic surgeon to an acute medical physician. The aim of this change was to ensure that all patients received a senior medical review within 4 hours of admission, with a medical consultant review within 12 hours, and subsequent orthogeriatric review within 24 hours. Other benefits of an acute medical admission included early identification and initiation of urgent medical therapy to treat potentially reversible medical conditions, a higher level of nursing support and care, and the availability of monitored beds, if required, which were not as easily available elsewhere. We report on our experience and outcomes following this change in practice.

## METHODS

A prospective audit of all adult patients admitted to our institution with a confirmed proximal femoral fracture between 1^st^ December 2010 and 31^st^ November 2011 was conducted.

### Inclusion and Exclusion Criteria

All patients admitted to our institution in the specified time frame with a proximal femoral fracture were included in the audit. This included patients with either an intracapsular (femoral neck) or extracapsular (trochanteric/subtrochanteric) proximal femoral fracture, regardless of age and mechanism of injury. Patients with diaphyseal femoral fractures or peri-prosthetic femoral fractures around a hip prosthesis were excluded from the audit. There were no other exclusion criteria.

### Data Collection

Data collection was carried out by EB and entered onto the NHFD. Data collected included:

- Pre-admission information: age, gender, level of accommodation, mobility indoors and outdoors.
- Admission details: date and time of presentation to A&E, time of admission to a ward, nature of admitting ward (surgical, orthopaedic, medical, other), abbreviated mental test score (AMTS, 0-10).
- Peri-operative details: date and time of orthogeriatric assessment, ASA grade, date and time of surgery, delays to surgery and reason(s) for these.
- Post-operative information: discharge date and destination, inpatient mortality.

### Audit Standards and Implementation

In the first audit cycle, 1^st^ December 2010 – 31^st^ May 2011, the audit standards were: (a) time to orthogeriatric assessment < 72 hours, and (b) time to surgery < 36 hours, as required by the BPT targets.

**Fig. (1):**
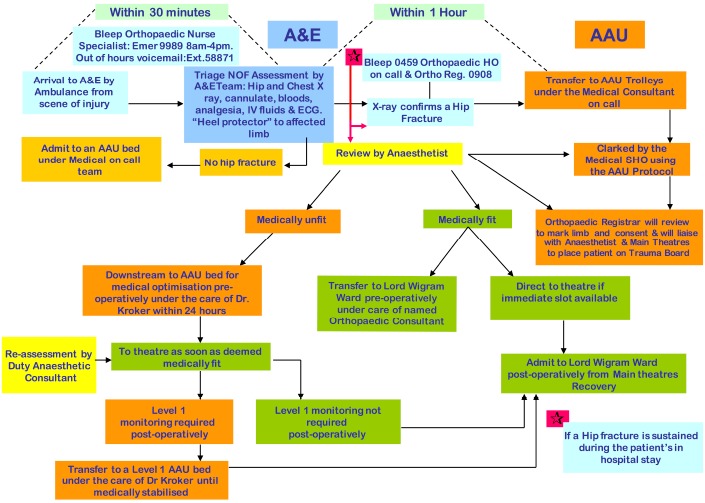
Hip Fracture Pathway, introduce in June 2011.

A new hip fracture pathway was implemented in our institution on 1^st^ June 2011 (Fig. **1**). This was based on NICE and BOA guidelines. Education of A&E staff, admitting medical, orthopaedic and anaesthetic junior doctors, and nursing staff on AAU was undertaken in the form of seminars and laminated posters. The main changes included:

1. Admission directly from the emergency department to the acute medical ward.
2. Joint care provided by a named on-call medical and orthopaedic consultant.
3. Full medical evaluation by the acute medical team, with the aim of diagnosing and correcting reversible problems prior to surgery.
4. Anaesthetic assessment within 12 hours of admission.
5. Consultant orthopaedic and orthogeriatric review within 24 hours of admission.
6. Patient transfer to orthopaedic ward when medically fit (usually day 1 post-operatively, unless patient requiring high-dependency or level 1 care).

To validate the impact of the pathway, the audit cycle was closed by conducting a review of patient outcomes between 1^st^ June – 30^th^ November 2011. The audit standards in the second cycle were: (a) 100% pre-operative medical assessment, (b) time to orthogeriatric assessment < 24 hours, and (c) time to surgery < 36 hours. These standards were based on the pathway introduced in our institution.

Other outcome measures of interest included reasons for delays to surgery of > 36 hours, length of inpatient hospital stay, discharge destination and inpatient mortality rate.

In both audit cycles, the choice of operation for each patient was determined at a consultant-led multi-disciplinary meeting, taking into account the patient’s fracture pattern, fitness for surgery, pre-morbid function and pain. All operations took place on a consultant supervised trauma list by appropriately experienced surgeons in training. All patients received appropriate post-operative care, led jointly by a consultant orthopaedic surgeon and a consultant orthogeriatrician.

### Statistical Analysis

Data was analysed using SPSS software. Significance was tested using Chi Squared, Fisher’s exact and unpaired Student t-Tests, with statistical significance set at p<0.05.

## RESULTS

A total of 161 consecutive patients were admitted with a proximal femoral fracture in the specified time period. 92 patients were admitted before the implementation of the pathway (December 2010 – May 2011), and 69 patients after (June – November 2011). The number of patients in each group who underwent surgery were 84 and 66 respectively. Patients not requiring operation were either cases of stable valgus-impacted intracapsular fractures that were mobilised successfully, or those with fractures deemed old, confirmed on subsequent imaging, and also mobilised successfully. Those patients were included in the final analysis. Table **1** shows the demographics in each group, with both groups equivalent in terms of age, male:female ratio, ASA grade and pre-operative AMTS.

In the six months after the introduction of the pathway, there was a significant increase in the proportion of patients who received a pre-operative medical assessment (19% before, 85% after, p=0.0001). The mean time to orthogeriatric assessment (TtG) was reduced significantly from 91 to 19 hours (p=0.0001), and the mean length of stay (LOS) was reduced from 24.8 to 19.5 days (p=0.029). A significant reduction in mortality of 10% (14% before *vs.* 4% after, p=0.0336) was found, as well as an increase in the proportion of patients returning to their original place of accommodation (57% before, 80% after, p=0.0069).

There was an observed reduction in mean time to surgery (TtS) of 6 hours (37 hours to 31 hours), although this did not reach statistical significance (p=0.0663). There was, however, a notable reduction in the proportion of patients cancelled or delayed due to inadequate preoperative medical optimisation, which was the cause in 41% of cases delayed > 36 hours before implementation of our pathway, and reduced to only 17% of delayed cases after. Our results are summarised in Table **2** and Fig. (**2**).

## DISCUSSION

The management of fragility fractures of the hip has become a major public health concern due to an aging population and increasing costs of social care [3]. Currently, the incidence of fragility hip fractures is 75,000 cases per year in the UK, at an annual cost of approximately £2 billion to treat when taking into account all of the medical and social care required [10]. Until effective primary prevention strategies are implemented to reduce the incidence of such injuries, we must become more innovative in our approach to dealing with the increasing burden of disease faced in our hospitals.

Anecdotally, we see widespread variability in how this is practically achieved. For many trusts, a direct admission to an orthopaedic ward is the usual pathway into the hospital, where an acute medical assessment only takes place if specifically requested by the admitting orthopaedic team. Learning from our own experience of using this more ‘traditional’ patient pathway, we found that patients were more likely to be cancelled on the day of surgery due to inadequate medical optimisation.

In our institution, we were able to achieve a consensus agreement to admit all patients with proximal femoral fractures to an acute medical ward directly from the emergency department, under a named acute medical physician, as well as an orthopaedic surgeon. The perceived benefit of structuring our pathway in this way was to ensure all necessary investigations and therapeutic interventions could be initiated in a more timely fashion, with the benefit of reducing the likelihood of surgical cancellations. Based on our data, this simple intervention directly led to improvements in our patients’ length of stay, in-patient mortality and level of independence.

All patients were subsequently reviewed by a dedicated orthogeriatric team. Presently, the NHFD expects an orthogeriatric assessment to take place within 72 hours of admission to achieve the BPT. We sought to improve on this by adapting our audit standards to ensure an orthogeriatric assessment took place within 24 hours. After our intervention, the proportion of patients receiving a pre-operative medical assessment significantly improved from 19% to 85%. This compares very favourably with the national average of 37% in 2011 and 43% in 2012, as reported in the NHFD National Report 2012 [11].

There is evidence showing that operative delays lead to increased length of hospital stay, morbidity and mortality [12, 13]. Whilst we were unable to demonstrate a significant reduction in time to surgery, we were able to significantly reduce the proportion of patients cancelled due to inadequate preoperative medical optimisation. In the second arm of this audit, the most common reason for breeching the 36-hour BPT target for time to surgery was a lack of available morning operating lists, with trauma lists in our unit traditionally scheduled in the afternoon on four out of five days of the working week. This has since been addressed by rescheduling lists to the morning session, helping to further reduce time to surgery.

Furthermore, a significant reduction in length of stay to a mean of 19.5 days (national average 21.2 days in 2011, 20.2 in 2012 [11]) and in patient mortality to 4% (national average 9.5 and 9.1 in 2011 and 2012 respectively [11]) emphasises the improvement in patient outcomes from this intervention.

The ultimate goal of hip fracture management is to allow return to baseline function as early and safely as possible. One of the key improvements noted from our work was the significant increase in the proportion of patients returning to their pre-morbid level of accommodation. Achieving this requires a multi-disciplinary approach to their medical assessment, treatment and rehabilitation, supported by a timely and safe discharge from hospital.

In our unit, the routine post-operative management of hip fracture patients, including thromboprophylaxis guidelines, pain management, nutritional supplementation, falls risk assessments, early mobilisation and rehabilitation remained constant during the two phases of our audit cycle. This suggests that our intervention of early medical optimisation on an acute medical ward was an independent factor in increasing patient return to pre-morbid accommodation. The cost implications of this are substantial. The total 1-year cost for a patient with a hip fracture, including the operation, is currently estimated at US $25,000 [14]. This cost can more than double if patients are transferred to long-term care instead of returning to their pre-morbid place of accommodation [15]. Furthermore, there are substantial personal and psychological advantages for patients being able to maintain their independence. Indeed, this may ultimately be the most effective way of reducing the overall cost of treating patients with hip fractures by reducing the costs associated with hospital and long-term residential or nursing-home care.

We already know that the implementation of the NHFD along with the introduction of BPT has significantly improved patient outcomes by ensuring a greater proportion of patients receive early orthogeriatric assessment, shorter time to surgery and reduced length of stay [11, 16]. In our institution, similar improvements have been achieved through a novel intervention focused primarily on early orthogeriatric, orthopaedic and anaesthetic input, with rapid medical optimisation and prompt surgery. As a result we have been better able to more effectively increase compliance with BPT requirements, leading to increased financial reward. Further improvements are still possible, particularly with regards to further reducing time to surgery and achieving 100% pre-operative orthogeriatric assessment.

We acknowledge the limitations of our study, including a relatively small patient sample and analysis of patient outcomes only until time of discharge. We also acknowledge that not all patients with proximal femoral fractures would be suitable for this pathway, *e.g.* polytrauma patients may require a higher level of care or different model of optimisation, and patients with pathological fractures may require liaison with tertiary centres.

Nevertheless we share our experience at a district general hospital in a semi-urban area of London, as we believe our pathway, which is simple to implement and confers no additional cost to the trust, has significantly and consistently improved the care we provide to our hip fracture patients. Where feasible and practically possible, we would encourage other units to adopt similar ideas and novel interventions to improve patient care and outcomes. Centres considering the implementation of a similar pathway should first perform a feasibility study to assess its impact on the provision of other acute services, including other acute medical admissions, and any additional training that might be required, such as additional medical unit nursing training on managing the orthopaedic aspects of patients’ care.

## CONCLUSION

Joint medical and orthopaedic care from the time of admission can lead to a reduction in length of stay and inpatient mortality and increase the proportion of patients returning to their original place of accommodation. This not only helps to reduce the demand on more costly forms of community-based care, our intervention has also improved our compliance with BPT, providing the financial opportunity to invest in and further develop this and other patient services.

## Figures and Tables

**Fig. (2):**
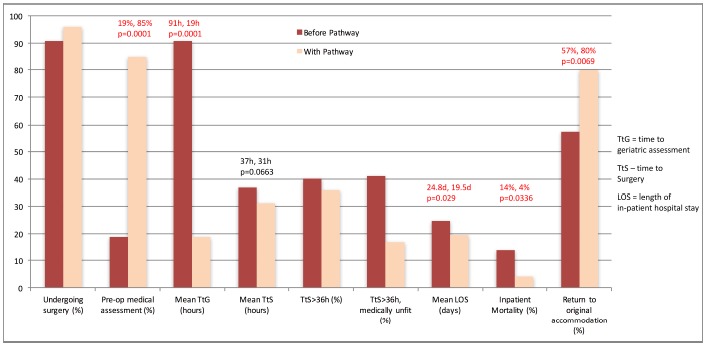
Graphic illustration of difference in result between first and second audit loops.

**Table 1:** Patient demographics in each group.

	**Before Pathway**	**With Pathway**	**p-value**
Total number of patients	92	69	
Requiring surgery	84	66	
Mean Age	79.8	76.9	0.1507
F:M ratio	2.5:1	2.6:1	0.9192
Mean AMTS	7.09	7.94	0.0815
Mean ASA grade	2.54	2.50	0.7325

**Table 2:** Outcomes before and after implementation of hip fracture pathway.

	**Before Pathway**	**With Pathway**	**p-value**
Total number of patients	92	69	
UUndergoing surgery	84	66	
Pre-op orthogeriatrician assessment	16/84 (19%)	56/66 (85%)	**0.0001**
Mean TtG (hours)	91 (SD140)	19 (SD19.3)	**0.0001**
Mean TtS (hours)	37 (SD32.6)	31 (SD16.5)	0.0663
TtS>36h	34/84 (40%)	24/66 (36%)	
TtS>36h, medically unfit	14/34 (41%)	4/24 (17%)	
Mean LOS (days)	24.8	19.5	**0.0290**
Inpatient Mortality	13/92 (14%)	3/69 (4%)	**0.0336**
Return to original accommodation	45/79 (57%)	53/66 (80%)	**0.0069**
